# Relevance of the GH-VEGFB/VEGFA axis in liver grafts from brain-dead donors with alcohol-associated liver disease

**DOI:** 10.3389/fcell.2024.1455258

**Published:** 2025-01-07

**Authors:** Marc Micó-Carnero, Carlos Rojano-Alfonso, Cristina Maroto-Serrat, Juan Carlos Cutrin, Araní Casillas-Ramírez, Carmen Peralta

**Affiliations:** ^1^ Department of Liver, Digestive System and Metabolism, Institut d'Investigacions Biomèdiques August Pi i Sunyer, Barcelona, Spain; ^2^ Universitat de Barcelona, Barcelona, Spain; ^3^ Molecular Biotechnology Center II “Guido Tarone”, Department of Molecular Biotechnologies and Science for the Health, University of Torino, Torino, Italy; ^4^ Hospital Regional de Alta Especialidad de Ciudad Victoria, IMSS-BIENESTAR, Ciudad Victoria, Mexico; ^5^ Facultad de Medicina e Ingeniería en Sistemas Computacionales de Matamoros, Universidad Autónoma de Tamaulipas, Matamoros, Mexico

**Keywords:** liver transplantation, brain death, ALD, ischemia-reperfusion, growth hormone, VEGF

## Abstract

**Introduction:**

Grafts with alcohol-associated liver disease (ALD) subjected to prolonged cold ischaemia from donors after brain death (DBD) are typically unsuitable for transplantation. Here, we investigated the role of growth hormone (GH) in livers with ALD from DBDs and its relationship with vascular endothelial growth factor A (VEGFA) and VEGFB.

**Methods:**

Livers from rats fed ethanol for 6 weeks and with brain death (BD) were cold stored for 24 h and subjected to *ex vivo* reperfusion. Hepatic damage and proliferative and inflammatory parameters were analysed after BD, before graft retrieval, and after reperfusion. Survival was monitored using an *in vivo* transplantation model.

**Results:**

In DBDs, the administration of GH, which increased the levels in the intestine but not in the liver, induced the generation of both VEGFA and VEGFB in the intestine and protected against hepatic damage caused by BD before retrieving liver grafts from donors. However, VEGFA was the only factor that protected against damage after cold ischemia and reperfusion, which also increased the survival of the recipients.

**Discussion:**

In conclusion, the signalling pathway and beneficial properties of the GH-VEGFA/VEGFB pathway, in which the intestine-liver axis plays a key role, were disrupted when grafts with ALD from DBDs were retrieved from donors and subjected to cold ischemia and reperfusion.

## 1 Introduction

Currently, more than 70% of grafts are obtained from donors after brain death (DBDs) (Kwong et al., 2021). Brain death (BD) markedly reduces liver graft tolerance to ischemia/reperfusion (I/R) injury as well as graft survival (Van Der Hoeven et al., 2001; ONT, 2023; Weiss et al., 2007). In clinical liver transplantation (LT), the shortage of hepatic graft donors and consequent increase in waiting lists for transplants has led centres to expand their criteria for accepting organs from marginal donors.

According to the National Transplant Organization (ONT), approximately 25% of grafts come from donors with some kind of alcoholic condition, and during the last 10 years, many of these liver grafts have been discarded because of the presence of alcohol-associated liver disease (ALD). Failure and dysfunction of liver grafts are directly related to the amount of alcohol consumed by donors throughout their lifetime (ONT, 2023). ALD has a high prevalence in society and represents a socioeconomic problem, and steatosis is a key donor variable for predicting post-transplant outcomes (ONT, 2023). ALD could be accompanied by steatosis and inflammation (alcoholic steatohepatitis, ASH) (Mackowiak et al., 2024). Its progression causes not only liver damage, but also the affectation of different tissues such as the intestine among others (Wu et al., 2023). Furthermore, it is known that excessive alcohol consumption can modulate the immune system by increasing or reducing its activity (Barr et al., 2016; Lewis et al., 2023; Wu et al., 2023). Globally, data indicate that alcohol causes 3 million deaths annually and is one of the major causes of morbidity and mortality related to liver pathologies (Wu et al., 2023). ALD in clinical liver transplants from DBD, especially when liver grafts are subjected to prolonged ischemic periods, such as 24 h, negatively affects postoperative outcomes and recipient survival (ONT, 2023; Pan et al., 2018).

Some studies suggest that endocrine abnormalities in DBDs include a rapid decrease in circulating hormones, such as growth hormone (GH) (Kgosidialwa et al., 2019; Kreber, Griesbach, and Ashley, 2016). GH is released from the anterior pituitary gland, circulates in plasma, and binds to its receptors in different target tissues (Álvarez-Mercado et al., 2019). Moreover it has been reported that GH can modulate pathways affected by ALD (Qin and Tian, 2010), thus establishing a relationship between GH, BD, and ALD.

Different results have been reported regarding the effects of GH on various pathologies. For example, GH replacement protects against inflammatory responses in cardiovascular diseases (Boccanegra et al., 2023; Caicedo et al., 2018). However, previous studies of LT from DBDs using a genetically induced obesity model of liver steatosis and without associated inflammation reported that steatotic livers subjected to 6 h of cold ischaemia (CI) had altered GH levels and an exacerbation of damage after exogenous GH administration (Álvarez-Mercado et al., 2019). In addition, preclinical ALD models have demonstrated that GH administration over long periods (6 weeks) may exert a positive effect on reducing fat infiltration (Qin and Tian, 2010).

GH is well known to have the ability to induce changes in different growth factors in liver pathologies. An *in vitro* model of a hepatocellular carcinoma (HCC) cell line showed that GH may increase vascular endothelial growth factor A (VEGFA) levels and promote proliferation (S. Li et al., 2010). In experimental models of genetically induced obesity, different results have been reported regarding the effects of VEGFA on I/R injury. VEGFA plays a minor role in the benefits of the NRG1-PAK1 signalling pathway in steatotic LT from DBDs (Micó-Carnero et al., 2022), whereas VEGFA exacerbates damage in steatotic livers undergoing partial hepatectomy (PH) under I/R (Bujaldon et al., 2019). Many studies, including those related to PH, have reported that VEGF receptor 2 (VEGFR2) is the principal mediator of the pathophysiological effects of VEGFA (Ding et al., 2010; Rossato et al., 2020; Zhang et al., 2019; Zhou et al., 2022).

Moreover, VEGFA and VEGFB are closely related, with VEGFA having affinity towards VEGFR2 and VEGFR1 (the VEGFB receptor) (Boucher et al., 2017; Lal, Puri, and Rodrigues, 2018). VEGFB plays an important role in fatty liver. In a recent study using diabetic and NAFLD-induced mouse models, treatment with anti-VEGFB for long periods (2 months) prevented NAFLD development by blocking white adipose tissue lipolysis (Falkevall et al., 2023). In contrast, results based on VEGFB-KO mice with induced obesity indicate that VEGFB increases NAFLD development (R. Li et al., 2022). To the best of our knowledge, studies on the role of VEGFB in hepatic I/R conditions have not been reported.

Given the close bidirectional anatomical and functional relationship between the gastrointestinal tract and the liver via portal circulation, several studies have reported an important role of the intestine in I/R liver injury (Micó-Carnero et al., 2021). It is also important to highlight the role of other factors in the importance of this axis in the context of LT, such as certain growth as FGF15 (Álvarez-Mercado et al., 2019; Gulfo et al., 2020). These data added to the fact that a recent study demonstrated the production of VEGFB and VEGFA in the intestine (Zhang et al., 2018), suggest that intestine-liver axis may be crucial in the study of those factors role.

Herein, we explored the role of GH, VEGFB, and VEGFA in the following conditions: (a) in DBDs before graft retrieval from donors, evaluating whether GH modulates VEGFA and VEGFB and the importance of the intestine-liver axis in such pathways due to the close anatomical and functional bidirectional interaction between the intestine and liver and its crucial role as a modulator of diverse liver pathologies and liver injury in I/R injury (Micó-Carnero et al., 2020); (b) in an *ex vivo* model after 24 h of cold ischaemia in a liver normothermic perfusion machine; and (c) in an LT model in order to study the postsurgical survival rate. Our investigations focused on the pharmacological modulation of GH, VEGFB, and VEGFA, as well as the involvement of the intestine-liver axis in DBDs before liver retrieval from donors, to investigate whether the effects on the liver induced by the pharmacological modulation of such growth factors (GH-VEGFB/VEGFA) in DBDs are maintained when liver grafts are retrieved from DBDs and subjected to 24 h of cold ischaemia in either an *ex vivo* liver perfused model or *in vivo* LT. The results of this study could lead to the identification of new molecular mechanisms and therapeutic targets that would address the unmet need of improving the function and quality of livers with ALD in LT from DBDs.

## 2 Material and methods

### 2.1 Animals and experimental model of ALD

Male Sprague-Dawley (SD) rats weighing 200–250 g were used. Rats were fed standard chow (Teklad Global 14% Protein Rodent Maintenance Diet; ENVIGO). ALD was induced *ad libitum* by diluting ethanol (12% v/v) in tap water for 6 weeks to induce chronic toxicity (Nevzorova et al., 2020; Radic et al., 2019). Steatosis (via Oil Red O staining), fibrosis (via Sirius red staining), and collagen I and alpha-smooth muscle actin (α-SMA) levels were measured. Low steatosis levels (<30% steatosis) and an increase in fibrotic markers were detected in the liver (Supplementary Figure S1). Rats were maintained under a 12-h light/dark cycle in a room at 25°C ± 2°C.

All procedures were conducted according to EU regulations (Directive 2010/63/EU of the European Parliament and Council of 22 September 2010).

### 2.2 Experimental groups

To study the effects of the GH pathway on hepatic damage in DBD livers after BD induction in an ALD experimental model, we established the following experimental groups:

1. Cont. 1 (n = 6): rats with ALD (see Section 2.1).
2. BD (n = 6): BD was performed via frontolateral trepanation in rats with ALD, and a balloon catheter was introduced into the extradural space. Intracranial pressure was increased by inflating the balloon for 1 minute, which induced rapid brain injury and led to immediate BD. The rats were maintained normotensive with colloid infusion for 3 h.
3. BD + GH (n = 6): as in group 2, with recombinant GH (100–40, Thermo Fisher Scientific, Waltham, Massachusetts, United States of America) administered intravenously immediately after BD (0.1 mg/kg).
4. BD + VEGFB (n = 6): as in group 2, with VEGFB (100-20B-100UG, Thermo Fisher Scientific, Waltham, Massachusetts, United States of America) administered intravenously immediately after BD (5 μg/kg).
5. BD + VEGFA (n = 6): as in group 2, with recombinant VEGFA (100–20-100UG, Thermo Fisher Scientific, Waltham, Massachusetts, United States of America) administered intravenously immediately after BD (5 μg/kg).
6. BD + GH + anti-VEGFR1 (n = 6): as in group 3, with an antibody against VEGFR1 (BS-0170R-100UL (Bioss Inc., Woburn, Massachusetts, United States of America) administered intravenously immediately after BD (10 mg/kg).
7. BD + GH + anti-VEGFR1 + anti-VEGFR2 (n = 6): as in group 6, with an VEGFR2 antagonist (orb61120, Biorbyt, Cambridge, United Kingdom) administered intravenously (2.5 mg/kg).

Tissue and plasma samples were collected 3 h after BD induction and immediately frozen at −80°C. Liver samples were fixed in 4% buffered formaldehyde solution overnight at 4°C and in OCT for histological and immunohistochemical analyses.

To investigate the effects of the GH pathway on hepatic damage in the experimental model after BD, cold ischaemia (CI) and reperfusion in our ALD experimental model, an *ex vivo* normothermic perfusion model, was established. After 3 h of BD, livers were flushed with University of Wisconsin (UW) solution, isolated, and preserved in UW solution at 4°C for 24 h. Livers were then connected via the portal vein to a recirculating perfusion system for 120 min at 37°C. In the *ex vivo* liver perfusion model, the perfusate was composed of 2% dextran in Krebs-Henseleit bicarbonate (KHB) buffer (K3753, Sigma Aldrich from St. Louis, Missouri, United States of America) with 2 µI/mL of heparin. The buffer was continuously ventilated with a 95% O_2_ and 5% CO_2_ gas mixture (Carnevale et al., 2019). The following groups were established:

8. BD + CI (n = 6): as in group 2, but livers were flushed with UW solution, isolated, and preserved at 4°C for 24 h of CI.
9. BD + GH + CI (n = 6): as in group 3, but livers were flushed with UW solution, isolated, and preserved at 4°C for 24 h of CI.
10. BD + VEGFB + CI (n = 6): as in group 4, but livers were flushed with UW solution, isolated, and preserved at 4°C for 24 h of CI
11. BD + VEGFA + CI (n = 6): as in group 5, but livers were flushed with UW solution, isolated, and preserved at 4°C for 24 h of CI
12. Cont 2 (n = 6): The livers of ALD rats were flushed with UW solution without CI or reperfusion.
13. BD + CI/R (n = 6): as in group, 2 but livers were flushed with UW solution, isolated, and preserved at 4°C for 24 h of CI and 2 h of normothermic *ex vivo* reperfusion.
14. BD + GH + CI/R (n = 6): as in group 3, but livers were flushed with UW solution, isolated, and preserved at 4°C for 24 h of CI and 2 h of normothermic *ex vivo* reperfusion.
15. BD + VEGFB + CI/R (n = 6): as in group 4, but livers were flushed with UW solution, isolated, and preserved at 4°C for 24 h of CI and 2 h of normothermic *ex vivo* reperfusion.
16. BD + VEGFA + CI/R (n = 6): as in group 5, but livers were flushed with UW solution, isolated, and preserved at 4°C for 24 h of CI and 2 h of normothermic *ex vivo* reperfusion.

Liver and perfusate samples were collected after 24 h of CI and 2 h after reperfusion and were immediately frozen at −80°C. Liver samples were fixed in 4% buffered formaldehyde solution overnight at 4°C and in OCT for histological and immunohistochemical analyses.

All doses of the compounds administered in this study were determined based on previous studies (Álvarez-Mercado et al., 2019; Atzori et al., 2022; Bujaldon et al., 2019; Micó-Carnero et al., 2022) and preliminary data obtained by our group.

Finally, to study whether the treatments played a role in the survival rate, LT groups were created, as follows:

17. BD + LT (n = 12, 6 transplantations): After 3 h of BD (as in group 2), livers from ALD rats were flushed with UW solution, isolated, and preserved in UW solution at 4°C for 24 h. Livers were implanted in healthy rats according to the cuff technique described by Kamada, with an anhepatic phase of 15–20 min (Kamada and Calne, 1979).
18. BD + GH + LT (n = 12, 6 transplantations): Same as group 17, but with GH (dose and pretreatment times similar to those of group 3).
19. BD + VEGFB + LT (n = 12, 6 transplantations): Same as group 17, but with VEGFB (dose and pretreatment times similar to those of group 4).
20. ALD + BD + VEGFA + LT (n = 12, 6 transplantations): Same as group 17, but with VEGFA (dose and pretreatment times similar to those of group 5).

After the surgical procedure, the survival of receptors was monitored for 14 days.

### 2.3 Biochemical determinations

Plasma transaminases (alanine aminotransferase [ALT]; GN41125, Gernon, Sant Joan Despí, Spain and aspartate aminotransferase [AST]; GN40125, Gernon, Sant Joan Despí, Spain) were measured using standard procedures. GH (E-EL-R3003), VEGFA (E-EL-R2603; Elabscience Biotechnology Co., Ltd., Wuhan, China), and VEGFB (MBS269676; MyBioSource, Inc., San Diego, CA, United States of America) levels in the liver and the entire wall thickness of the small intestine were determined using an immunoassay kit. IL-1β (ab100768), collagen-I (ab285314; Abcam, Cambridge, UK), IL-10 (E-EL-R0016; MyBioSource, Inc., San Diego, CA, United States of America), Ki-67 (MBS705024) and α-SMA (MBS266620; MyBioSource, Inc., San Diego, CA, United States of America) levels were quantified in liver tissue with immunoassay kits, according to the manufacturer’s instructions. Lipid peroxidation was determined by measuring the formation of malondialdehyde (MDA) with the thiobarbiturate reaction (Casillas-Ramírez et al., 2023).

Caspase-3 (ab39401; Abcam, Cambridge, UK), ATP (MBS166244; MyBioSource Inc., San Diego, CA, United States of America), and lactate (E-BB-K044-S; Elabscience Biotechnology Co., Ltd., Wuhan, China) levels were measured using colorimetric assay kits. The total protein concentration in different tissues was determined using a colorimetric kit and the Bradford method (5000006; Bio-Rad, Hercules, CA, United States of America), according to the manufacturer’s instructions. Biochemical results were adjusted per mg of tissue protein.

### 2.4 Reverse transcription and quantitative polymerase chain reaction

Total RNA was isolated from frozen intestinal tissues using TRIzol Reagent (15596026, Invitrogen, Madrid, Spain), quantified with a NanoDrop 1,000 spectrophotometre, and reverse-transcribed using a High-Capacity cDNA Reverse Transcription Kit (4,374,966; Thermo Fisher Scientific, Life Technologies, Carlsbad, CA, United States of America). qPCR was performed with TaqMan Universal PCR Master Mix (4,304,437; Thermo Fisher Scientific, Life Technologies, Carlsbad, CA, United States of America) using an ABI PRISM 7900 HT detection system with premade Assays-on-Demand TaqMan probes (Rn01511602_m1 for VEGFA, Rn01454585_g1 for VEGFB, and Rn00667869_m1 for β-actin, as an endogenous control; Thermo Fisher Scientific, Waltham, MA, United States of America), according to the manufacturer’s protocol.

### 2.5 Histology and immunohistochemistry

To evaluate the severity of hepatic injury, paraffin-embedded liver sections were stained with haematoxylin and eosin (H&E). As an assessment parameter for liver injury, the sinusoidal status was evaluated, as described by Behrends et al. (2010), and sections were scored from 0 to 4 for sinusoidal dilatation, sinusoidal congestion, atrophy, perisinusoidal fibrosis, portal fibrosis, and portal inflammation.

Liver steatosis was evaluated by Oil Red O staining of the liver tissues in OCT sections. The TUNEL assay kit was used to identify apoptotic cells (nuclei or apoptotic bodies) using a DNA fragmentation detection kit (ab206386, Abcam, Cambridge, UK), and sections were dewaxed according to the manufacturer’s instructions. Finally, the fibrosis level was assessed by Sirius red staining to observe collagen I fibres.

Liver sections were observed under an Olympus BX51 System Microscope (Olympus, Tokyo, Japan).

### 2.6 Statistics

Statistical analyses were performed using Prism 10.0.2 for Windows (GraphPad Software, San Diego, California, United States of America). All results are expressed as mean ± standard error of the mean (SEM). The results were compared using one-way analysis of variance (ANOVA) with Tukey’s *post hoc* test. The Student’s t-test was performed for comparisons between two groups. Survival was estimated using the Kaplan-Meier method and statistically analysed using a log-rank test. Differences were considered significant at p-values-<0.05.

## 3 Results

### 3.1 GH-VEGFB/VEGFA pathway in DBDs with ALD

**FIGURE 1:**
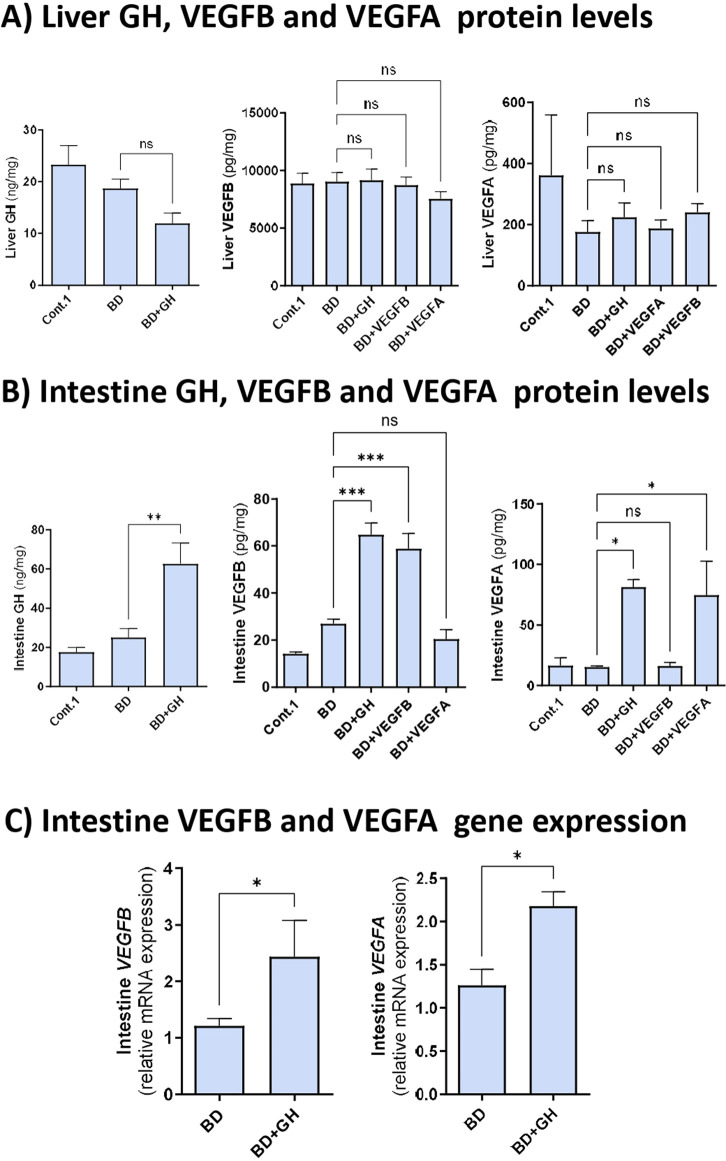
GH-VEGFB/VEGFA pathway in DBDs with ALD. **(A)** Liver GH, VEGFB and VEGFA protein levels. **(B)** Intestine GH, VEGFB and VEGFA protein levels. **(C)** Intestine VEGFB and VEGFA relative mRNA expression. ****p ≤ 0.0001; **p ≤ 0.01; *p ≤ 0.05 and ns = no significance.

First, to evaluate the role of this pathway, we determined the levels of GH and its effects on VEGFB and VEGFA in liver grafts after BD before graft retrieval. No significant between-group differences were observed in any of the protein levels (Figure 1A). Thus, under these experimental conditions, the exogenously administered GH, VEGFB, or VEGFA did not reach the liver. Indeed, the hepatic levels of GH in the BD + GH group were similar to those in the BD group. Similarly, liver levels of VEGFB and VEGFA after exogenous administration (BD + VEGFB and BD + VEGFA groups, respectively) were similar to those in the BD group.

Owing to the importance of the intestine-liver axis, we evaluated GH, VEGFB, and VEGFA levels in the intestines of DBDs before graft retrieval. The increase in GH observed in the intestine after GH administration (BD + GH group) was associated with an increase in the protein and mRNA expression of VEGFB and VEGFA (Figures 1B,C), indicating that GH administration promoted the intestinal production of VEGFB and VEGFA. Finally, in contrast to the liver, increases in VEGFB and VEGFA protein levels were detected in the intestine after exogenous administration (Figure 1B). Thus, contrary to what occurred in the liver, all factors (GH, VEGFB, and VEGFA) are taken up by the intestine (but not by the liver) after their exogenous administration.

To examine the potential co-regulation between VEGFB and VEGFA, VEGFB and VEGFA levels were evaluated in the liver and intestine. VEGFB levels were similar between the BD and BD + VEGFA groups, and VEGFA levels were similar between the BD and BD + VEGFB groups (Figures 1A,B).

### 3.2 Role of the GH-VEGFB/VEGFA pathway in DBDs with ALD before graft retrieval

In this study, we assessed the role of GH and its downstream effectors, VEGFB and VEGFA, in DBDs with ALD and evaluated their effects on liver damage, inflammation, oxidative stress, regeneration, cell death, and cell energy metabolism biomarkers before liver graft retrieval.

**FIGURE 2:**
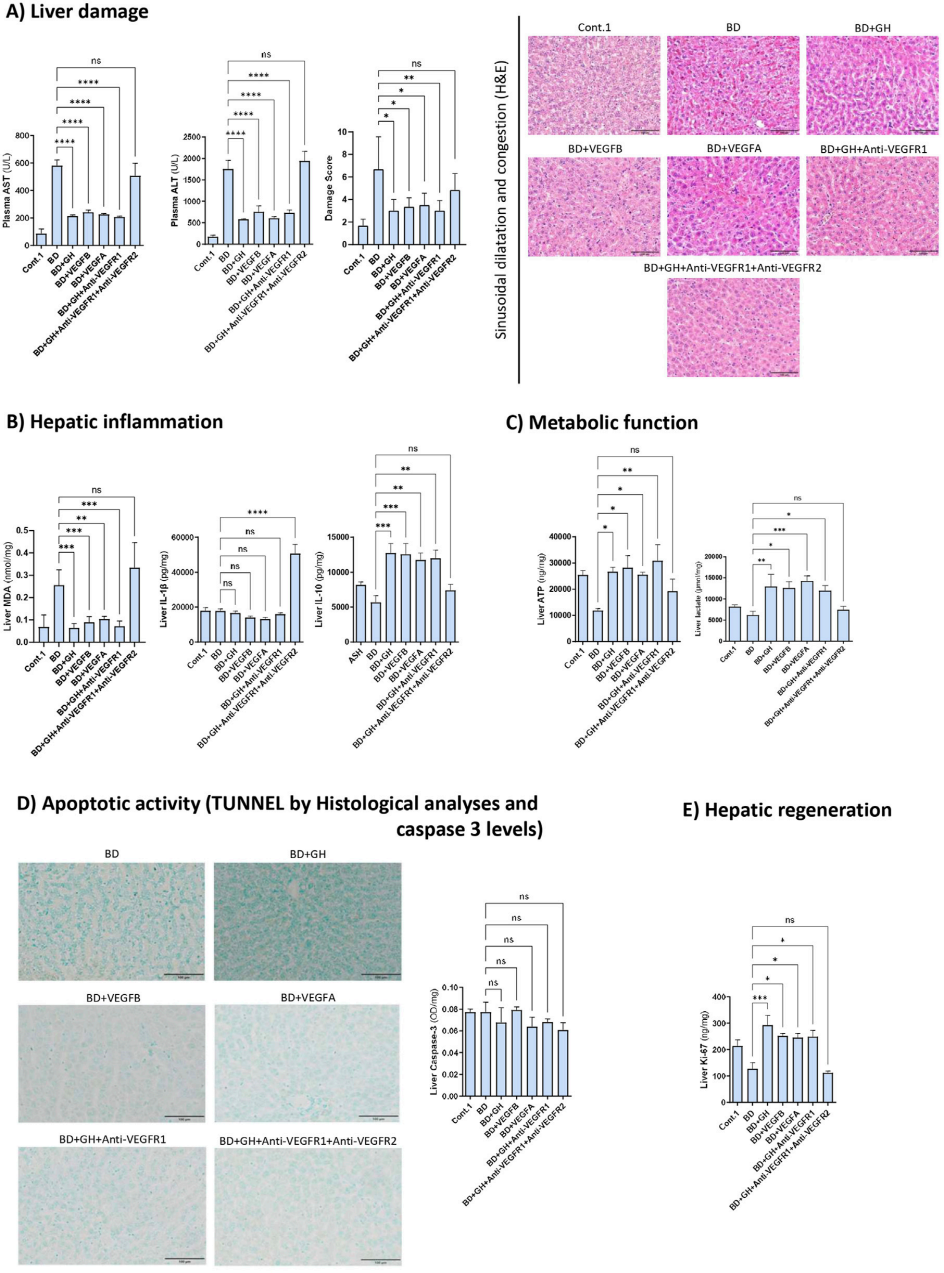
Relevance of GH-VEGFB/VEGFA axis in liver damage, inflammation, metabolic function cell death and regeneration in DBDs with ALD. **(A)** AST and ALT levels in plasma and liver damage score with representative photomicrographs of sinusoidal dilatation and congestion (20X) (scale bar corresponds to 100 µm). **(B)** Hepatic levels of MDA, IL-1β and IL-10. **(C)** Hepatic levels of ATP and lactate. **(D)** TUNEL stained sections (20X) to detect apoptotic cells (scale bar correspond to 100 µm) and caspase-3 liver levels. **(E)** Regeneration biomarker Ki-67 liver levels. ****p ≤ 0.0001, ***p ≤ 0.001, **p ≤ 0.01; *p ≤ 0.05 and ns = no significance.

A decrease in all liver damage parameters (AST and ALT levels and damage scores) was detected after administering GH, VEGFB, or VEGFA (Figure 2A). In addition, the concomitant administration of recombinant GH and anti-VEGFR1 (an antibody against the VEGFB receptor) protected ALD livers against the deleterious effects induced by BD (Figure 2A). However, when we concomitantly administered GH and inhibited the action of both receptors VEGFR1 and VEGFR2 (BD + GH + anti-VEGFR1 + anti-VEGFR2), the hepatic damage results were similar to those of the BD group.

Inflammation parameters were also analysed (MDA, IL-1β, and IL-10 levels). MDA levels (an index of oxidative stress) followed a pattern similar to that of hepatic damage; a decrease in MDA levels was observed after treatment with GH, VEGFB, VEGFA, and GH + anti-VEGFR1, whereas the GH benefits were abolished when both VEGFR1 and VEGFR2 were inhibited (the BD + GH + anti-VEGFR1+anti-VEGFR2 group) (Figure 2B). Inflammation parameters were also analysed. Considering the potential inflammatory effects of IL-1β, its levels were also assessed. Despite no significant differences between the different potential beneficial treatments and the BD group, a high increase in their liver levels was detected in the BD group treated with GH, anti-VEGFR1, and anti-VEGFR2 (the BD + GH + anti-VEGFR1+anti-VEGFR2 group), indicating the strong inflammatory potential of this pathway and a synergism in the inflammatory response of these modulators (Figure 2B). Next, we evaluated the potential anti-inflammatory effects of these pathways. A significant increase in IL-10 (a potent anti-inflammatory interleukin) was detected in the groups that were protected against hepatic damage (GH, VEGFB, VEGFA, and GH + anti-VEGFR1 groups), whereas hepatic IL-10 levels in the BD + GH + anti-VEGFR1+anti-VEGFR2 group were similar to those in the BD group (Figure 2B).

Subsequently, parameters related to energy metabolism (ATP and lactate) were analysed in ALD livers from DBDs. Effective treatments promoted anaerobic glycolysis to increase ATP. Indeed, high levels of lactate were directly related to high ATP levels in the liver in the BD + GH, BD + VEGFB, BD + VEGFA, and BD + GH + anti-VEGFR1 groups, when compared with the BD group (Figure 2C). However, ATP and lactate levels were similar in the BD + GH + anti-VEGFR1+anti-VEGFR2 and BD groups.

Finally, the apoptotic activity and hepatic regeneration were evaluated. To determine whether cell death occurred via apoptosis, the caspase-3 activity was evaluated. No significant differences were detected between the groups (Figure 2D). To confirm these data, liver sections stained with the TUNEL assay kit were evaluated, and no differences in the number of apoptotic cells were detected between the groups (Figure 2D). These data suggest that regulation of cell death by apoptosis does not play a relevant role in such conditions. Concerning the capacity of the GH-VEGFB/VEGFA axis for hepatic regeneration, Ki-67 liver levels were analysed. Livers in the BD + GH, BD + VEGFB, BD + VEGFA, and BD + GH + anti-VEGFR1 groups had increased Ki-67 levels compared with the levels in the BD group. Administering GH with anti-VEGFR1 and anti-VEGFR2 (the BD + GH + anti-VEGFR1 + anti-VEGFR2 group) resulted in Ki-67 values that were similar to those in the BD group (Figure 2E).

### 3.3 Role of GH, VEGFB, and VEGFA in liver grafts from BD donors with ALD after CI and reperfusion

Considering the previously obtained data, we assessed the role of GH, VEGFB, and VEGFA in ALD livers from DBDs after CI and reperfusion in an *ex vivo* model and survival studies.

**FIGURE 3:**
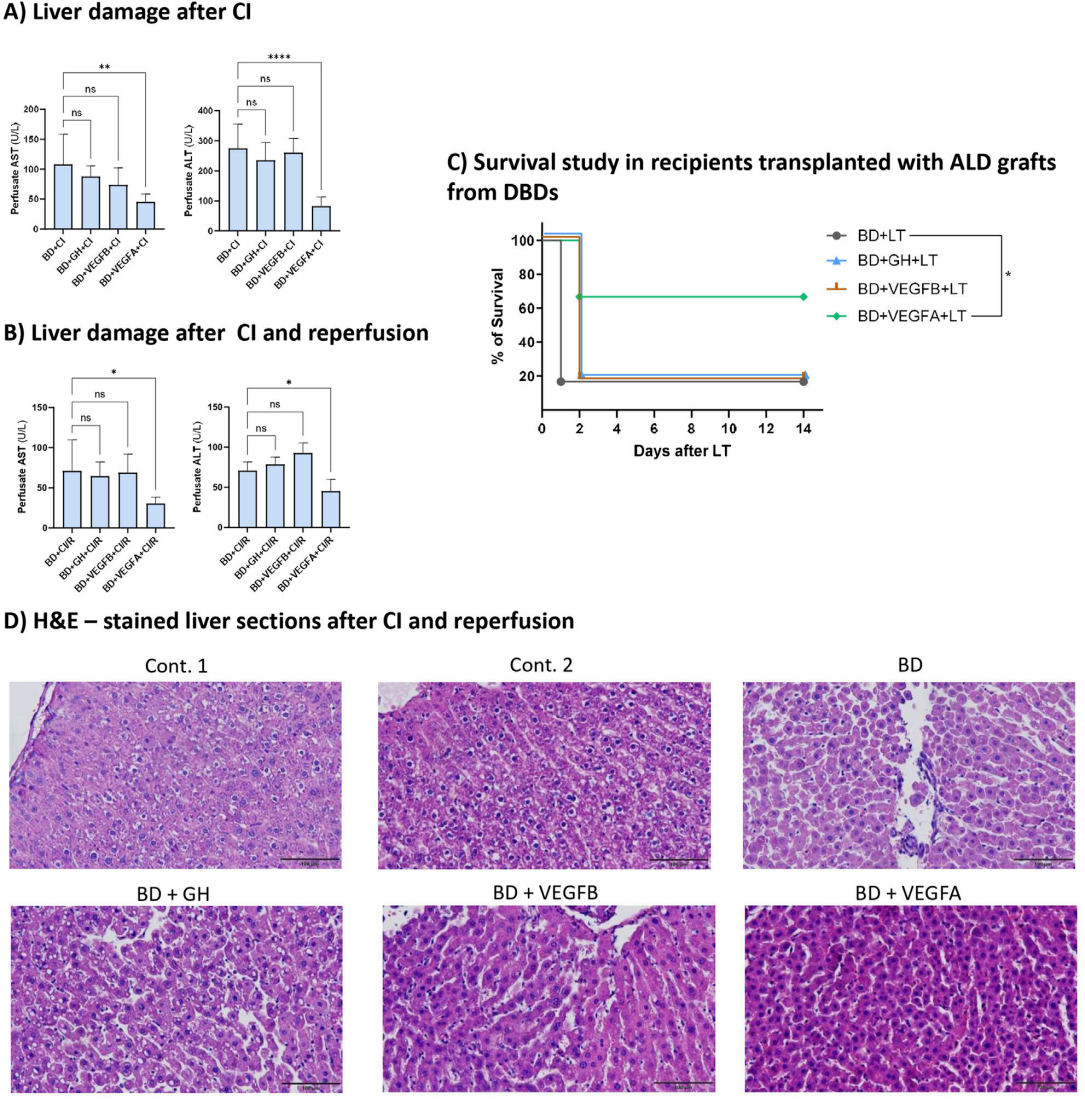
Effects of GH-VEGFB/VEGFA axis on liver damage and survival after CI or I/R injury. **(A)** AST and ALT levels in perfusates after 24 h of CI. **(B)** AST and ALT levels in perfusates after 24 h of CI and 2 h of reperfusion. **(C)** Survival studies of different recipients with ALD-grafts from DBDs. **(D)** H&E staining of liver sections after I/R injury at 20X (scale bar correspond to 100 µm). ****p ≤ 0.0001, ***p ≤ 0.001, **p ≤ 0.01; *p ≤ 0.05 and ns = no significance.

Contrary to the results obtained for liver grafts from DBDs and ALD, cold ischaemia affected the benefits observed for GH, VEGFB, and VEGFA in liver grafts with ALD from DBDs before their retrieval from donors. Indeed, our results indicated that the effectiveness of either GH or VEGFB before liver retrieval from donors was not observed when such liver grafts were submitted to 24 h of CI, and similar results were obtained after 24 h of CI followed by 2 h of normothermic reperfusion. For instance, the AST and ALT levels in the perfusate of the BD + GH + CI and BD + VEGFB + CI groups were similar to those in the BD + CI group (Figure 3A). Similarly, the AST and ALT levels in the perfusates of the BD + GH + CI/R and BD + VEGFB + CI/R groups were similar to those in the BD + CI/R group (Figure 3B). In contrast, the benefits of VEGFA administration in ALD liver grafts before their retrieval from DBDs were maintained after 24 h of CI and also after 24 h of CI followed by 2 h of normothermic reperfusion. This was reflected in the reduction in transaminase levels observed in perfusates obtained after 24 h of CI or 24 h of CI followed by 2 h of reperfusion (BD + VEGFA + CI and BD + VEGFA + CI/R groups) when compared to the results of the BD + CI and BD + CI/R groups, respectively (Figures 3A,B). These results are in accordance with the histological and morphological alterations observed in H&E-stained liver sections. Indeed, a considerable disorganized parenchyma and more sinusoidal alterations were observed in livers from the BD + CI/R, BD + GH + CI/R, and BD + VEGFB + CI/R groups, as compared with those of the BD + VEGFA + CI/R group (Figure 3D). Survival studies in an *in vivo* LT model demonstrated the same beneficial effects of VEGFA as those of GH or VEGFB. Recipients implanted with an ALD graft subjected to 24 h of CI from DBDs and treated with VEGFA (BD + VEGFA + LT group) had a survival rate of 67% at 14 days after surgery, whereas the survival of recipients treated with VEGFB, GH, or without treatment was reduced (17% for recipient survival) (Figure 3C).

**FIGURE 4:**
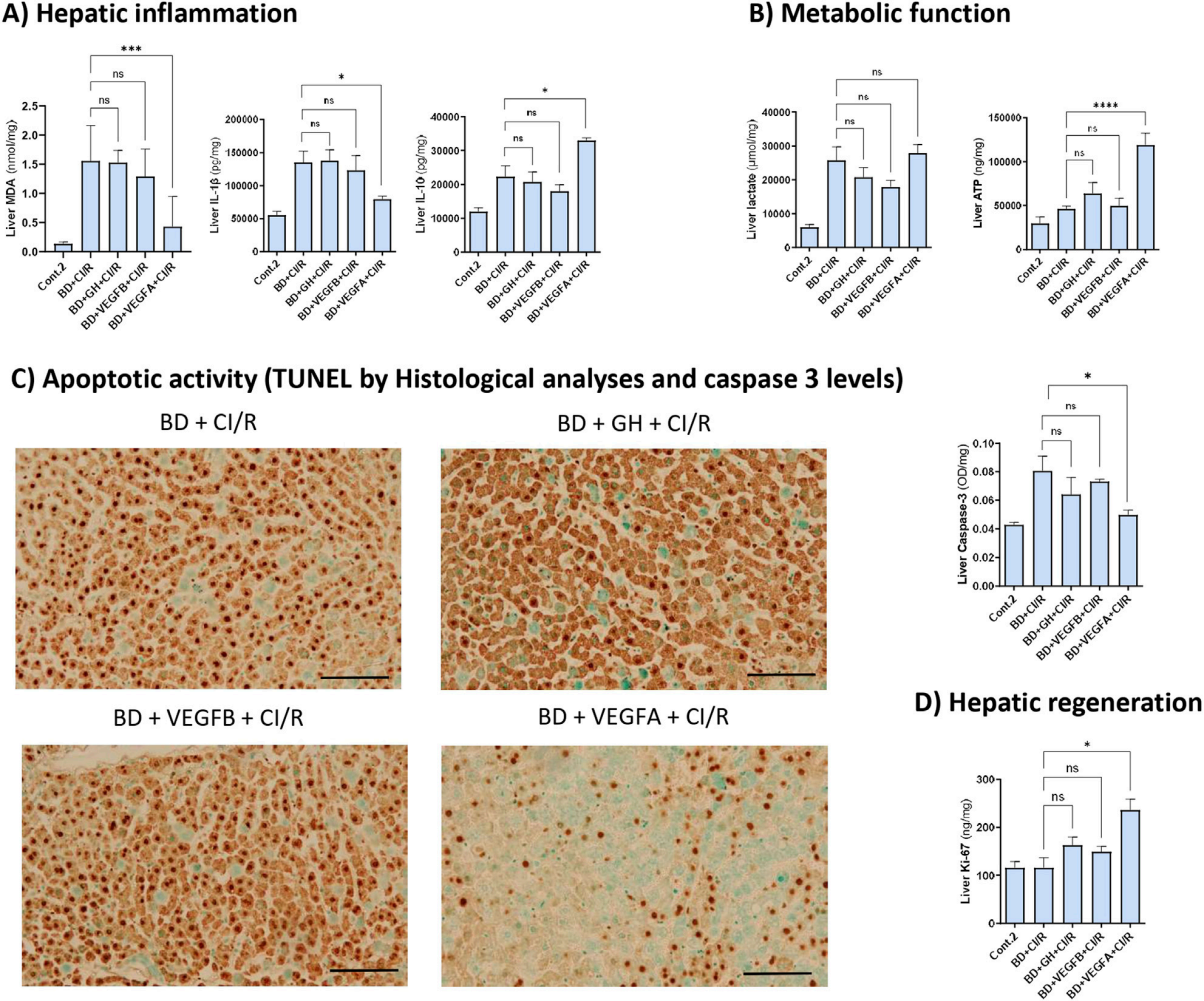
GH-VEGFB/VEGFA pathway importance on inflammatory, metabolic apoptotic and regenerative parameters after I/R injury. **(A)** Hepatic levels of MDA, IL-1β and IL-10. **(B)** Hepatic levels of ATP and lactate. **(C)** TUNEL stained sections (20X) to detect apoptotic cells or bodies (in brown) (scale bar correspond to 100 µm) and caspase-3 liver levels. **(D)** Regeneration biomarker Ki-67 liver levels. ****p ≤ 0.0001, ***p ≤ 0.001; *p ≤ 0.05 ns = no significance.

We also evaluated the inflammatory response in liver grafts after CI/R injury, and the same results as those obtained for liver damage after CI/R injury were observed (Figure 4A). High MDA and IL-1β levels were maintained in grafts from DBDs treated with GH or VEGFB (BD + GH + CI/R and BD + VEGFB + CI/R groups), showing oxidative stress and inflammatory activity in those grafts, and was only diminished in the livers of rats administered VEGFA (BD + VEGFA + CI/R group), indicating the benefits of VEGFA on IL-1β levels. Finally, the levels of IL-10 (an IL with anti-inflammatory properties) were increased only in the livers of the BD + VEGFA + CI/R group (Figure 4A), indicating the activation of anti-inflammatory pathways in this group after CI/R.

Regarding metabolic parameters, liver ATP and lactate levels were analysed (Figure 4B). While lactate levels were unaltered, liver ATP levels were in concordance with inflammatory or liver damage parameters after CI/R in ex vivo-perfused livers, whereas the group with less liver damage (BD + VEGFA + CI/R group) had increased liver ATP levels. This indicated an increase in ATP levels due to the activation of aerobic metabolism or lower ATP depletion.

Finally, we evaluated apoptotic activity and regeneration after CI/R. In the grafts of the groups with higher liver damage parameters (BD + CI/R, BD + GH + CI/R, and BD + VEGFB + CI/R groups), the apoptotic activity remained high. This was confirmed by assessing the caspase-3 activity and TUNEL staining (Figure 4C). Only a decrease in cell death due to the activation of apoptotic pathways was detected in the group with less liver damage (BD + VEGFA + CI/R) (Figure 4C). Regenerative failure was similar between the BD + CI/R, BD + GH + CI/R, and BD + GH + VEGFB groups, whereas better regenerative ability (assessed by Ki-67 levels in the liver) was detected in grafts of the BD + VEGFA + CI/R group, compared to those of the BD + CI/R group (Figure 4D).

## 4 Discussion

Herein, we report, for the first time, a new signalling pathway involved in LT with ALD from DBDs involving GH and VEGFB/VEGFA, with specific actions depending on the different stages involved in LT from DBDs.

Our results indicate insignificant alterations in the hypothalamic-pituitary axis in DBD. Indeed, BD did not induce changes in GH levels in either the liver or intestine. GH is involved in numerous metabolic disturbances and liver pathologies (Fang et al., 2019; Rufinatscha et al., 2018; Sarmento-Cabral et al., 2021; Tateno et al., 2011), and according to our results, the administration of GH, which only reaches the intestine, improves the quality of liver grafts from DBDs with ALD before graft retrieval because it modulates VEGFB and VEGFA expression in the intestine. Treatment with GH, VEGFB, or VEGFA does not affect the liver, but interestingly protects the liver with ALD from BD-induced damage before liver retrieval from donors. However, the benefits observed with GH, VEGA, and VEGFB before liver retrieval from DBDs are different from those observed when liver grafts are retrieved and subjected to *ex vivo* and *in vivo* models of LT, as explained below.

GH may have a positive role in ALD. Thus, prolonged GH administration (6 weeks) is a promising approach to reduce fatty infiltration (Qin and Tian, 2010), and the levels of GH are correlated with alcohol consumption (Trifunović et al., 2016; Vatsalya et al., 2012). The same happens with VEGFA and VEGFB in livers from rodents fed with ethanol (Ceccanti et al., 2012; Costa et al., 2017). According to our results, the effects of GH when rats with ALD are subjected to LT from DBDs depends on the stage of the procedure. Thus, GH exerts benefits in liver grafts from DBDs and in the presence of ALD only in donors before retrieval of liver grafts from donors, whereas this protection disappears when such liver grafts are retrieved from donors and subjected to 24 h of CI followed by either 2 h of normothermic reperfusion (*ex vivo* model) or *in vivo* LT. Thus, this strategy seems to be inappropriate in LT with ALD subjected to 24 h of CI from DBDs.

To the best of our knowledge, only one study has established a relationship between GH and VEGFA; in a human cancer cell line, GH may upregulate VEGFA levels (S. Li et al., 2010). However, no study has reported a relationship between GH and VEGFB. Therefore, owing to the similarities in the mechanisms of action and receptors of both growth factors, we hypothesised that GH may upregulate both VEGFB and VEGFA. The hypothesis was based on the results of Ran et al., who found that the gastrointestinal tract of an animal model expresses the growth hormone receptor (GHR) (Ran et al., 2016), which could explain the action of GH in the intestine and liver. In addition, a recent study demonstrated the production of both factors (VEGFB and VEGFA) in the intestine (F. Zhang et al., 2018). According to the results presented here, the exogenous administration of GH in DBDs with ALD increased the intestinal production of VEGFB and VEGFA.

Under PH + IR conditions, VEGFA exacerbated hepatocellular damage in steatotic livers in an experimental model of genetically induced obesity (Bujaldon et al., 2019). In other studies, VEGFB has been described as a modulator of I/R injury in the heart (Kivelä et al., 2014; Raissadati et al., 2017). According to our results, the administration of GH induced VEGFB expression in the intestine, which dampened the hepatic damage in DBDs with ALD before liver graft retrieval from donors. VEGFA and VEGFB reduce liver injury in DBDs before graft retrieval from donors. Nevertheless, when the anti-VEGFR1 antibody was administered in combination with GH, the beneficial properties induced by GH in the liver were not reversed. The beneficial effects of GH were revoked only by the concomitant administration of an inhibitor of VEGFR2 (the VEGFA receptor). These results are consistent with the existing literature on the dynamics of the VEGFR1 and VEGFR2 signalling pathways. Many studies suggest that VEGFA can bind to both VEGFR1 and VEGFR2 but only exerts its function through VEGFR2, whereas VEGFR1 would be a “decoy.” VEGFB binding to its receptor (VEGFR1) displaces VEGFA to VEGFR2, thereby promoting the action of the second factor. The potential benefit of VEGFB is its ability to enhance the action of VEGFA (Boucher et al., 2017; Lal et al., 2018). This seems to occur in DBDs with ALD before the liver grafts are retrieved from the donors. Exogenous GH may increase VEGF, VEGFA, and VEGFB levels. In such cases, VEGFB binds to its receptor (VEGFR1), potentiating the binding of VEGFA to VEGFR2. When only VEGFA is administered, it bound to both VEGFR1 and VEGFR2, thus exerting its effects on VEGFR2. However, when VEGF-B is administered, it binds to VEGFR1, allowing endogenous VEGFA to bind to VEGFR2. When GH and anti-VEGFR1 are administered intravenously, the VEGFA produced by GH binds to VEGFR2, thereby exerting its function. Finally, when GH is administered with anti-VEGFR1 and anti-VEGFR2, neither VEGFA nor VEGFB bind to VEGFR1/2, and VEGFR2 does not exert survival or proliferation effects. According to our results, VEGFA and VEGFB may indirectly protect the liver by exerting their functions in the intestine because their levels increased in the intestine but not in the liver tissue after their administration or production. This can be explained by the intestinal expression of VEGFR1 and VEGFR2 (Wejman et al., 2013; Zhou et al., 2023). In this case, exogenous GH throughout the production of endogenous VEGFA and VEGFB in the intestine or exogenous VEGFA and VEGFB (which are taken up by the intestine and not by the liver), may exert beneficial effects by modulating different pathways and producing different mediators in the intestine, which could be released into the portal circulation and then taken up by the liver to avoid liver damage produced in DBDs with ALD. Multiple pathways are modulated by the VEGF family, including survival, apoptosis and cell death, proliferation, hypertrophic processes, and permeability or cell migration (Li et al., 2012; Melincovici et al., 2018; Shen et al., 2018). This hypothesis should not be discarded, but further investigations (not part of the current study) are required to address this issue.

According to our results, the effects of GH, VEGFB, and VEGFA differ when liver grafts are retrieved from DBDs with ALD and subjected to 24 h of CI and reperfusion in *ex vivo* and *in vivo* LT models. Indeed, changes in the effects of such growth factors have been observed after CI (before normothermic reperfusion or implantation of liver grafts in the recipient). Our preliminary results (data not shown) indicated that neither GH, VEGFB, nor VEGFA protein levels were altered during CI or CI/R injury, with levels similar to those of the Cont2 group. These data suggest that CI and reperfusion trigger or inhibit different signalling pathways that might counteract the benefits of either GH or VEGFB. Under our conditions, the effects of the evaluated signalling pathway (e.g., GH-VEGFB/VEGFA) might be very different depending on the stage of LT in which they are being evaluated. This is not surprising, because the mechanisms involved in BD might be completely different from those involved in CI and reperfusion. Although the aim of this study was not to explain the reasons for the differential effects of GH and VEGFB depending on the stage of LT, different hypotheses should be proposed. For instance, VEGFB, a growth factor derived from the generation of GH in the intestine by different signalling pathways, may promote NO production in the liver (Bobic et al., 2014). NO has opposite effects, depending on the liver type and microenvironment (Carrasco-Chaumel et al., 2005). According to a previous study, NO might exert benefits in the presence of low reactive oxygen species (ROS) concentrations but might exacerbate liver damage in the presence of high ROS concentrations. According to our results, ROS production was higher after CI/R than after 3 h of BD in liver grafts, which might explain the differential role of VEGFB in DBDs before liver retrieval and after CI/R injury. Moreover, given the results of the current study, the mechanisms that cause liver damage in DBDs before liver retrieval and after CI//R injury are different (e.g., apoptosis). Our results show that the apoptotic activity represented by the caspase-3 assay and TUNEL staining depends on the different LT stages. In fact, hepatic cells do not die by apoptosis in liver grafts in DBDs before their retrieval from donors, and apoptotic activity (high caspase-3 activity and TUNEL staining) was detected only after CI/R injury. Finally, the intestine-liver axis in DBD with ALD (where VEGFA and VEGFB are synthesised by GH in the intestine) is not present when liver grafts are retrieved from donors and submitted to 24 h of CI and subsequent reperfusion (in an *ex vivo* model) or implanted in a recipient (healthy and without ALD) in which the intestinal characteristics and the relationship between the intestine and liver might significantly differ.

**FIGURE 5:**
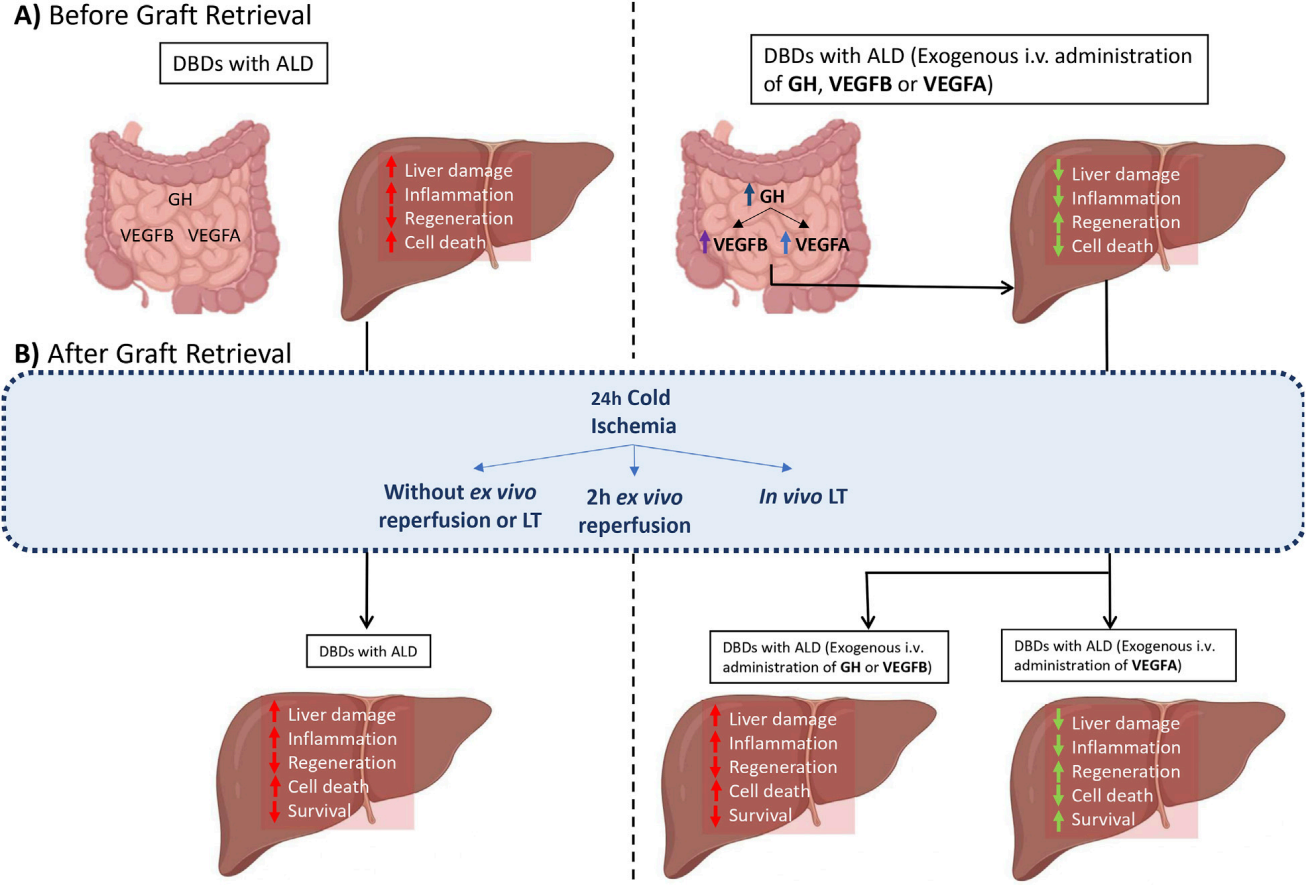
Schematic representation of GH-VEGFB/VEGFA axis in liver grafts from DBDs with ALD before and after graft retrieval. **(A)** Before Graft Retrieval. **(B)** After Graft Retrieval.

In conclusion, the results presented in this study demonstrate the relevance of VEGFB/VEGFA synthesised in the intestine by GH in LT from BD donors with ALD. GH may reduce the liver damage caused by BD before graft retrieval through the upregulation of both VEGFB and VEGFA in the intestine, whereas only VEGFA is feasible to reduce liver damage in DBDs with ALD after CI/R in experimental models of *ex vivo* and *in vivo* LT (Figure 5). This is of clinical and scientific interest because it is the first study to explore a potential therapy (namely, VEGFA treatment) to reduce the deleterious effects of BD and those induced by CI/R injury in liver grafts from DBDs with ALD.

## Data Availability

The original contributions presented in the study are included in the article/Supplementary Material, further inquiries can be directed to the corresponding authors.

## Supplementary material

SUPPLEMENTARY FIGURE S1 Liver histological sections and fibrosis biomarkers for ALD model. **(A)** Photographs of histological changes in liver sections from Healthy and ALD rats of: collagen-I fibers (white arrows) with Sirius Red staining at 10X and 20X in the amplified image (scale bar of 10X images corresponds to 200 µm and scale bar of 20X image corresponds to 50 µm); lipid droplets (black arrows) with Red Oil staining at 20X and 40X in the amplified image (scale bar of 20X images corresponds to 100 µm and scale bar of 40X image corresponds to 25 µm). **(B)** Liver α-SMA and collagen-I levels. *****p ≤ 0.0001 and ***p ≤ 0.001*.

SUPPLEMENTARY TABLE S1 Background of donors from whom the liver is implanted. Spain 2022. (Extracted and translated from ONT 2023).

SUPPLEMENTARY TABLE S2 Transplant percentage of removed livers according to donor characteristics. Spain 2013-2022. (Extracted and translated from ONT 2023).

## References

[B1] Álvarez-Mercado A. I. Negrete-Sánchez E. Gulfo J. Ávalos de León C. G. Casillas-Ramírez A. Cornide-Petronio M. E. (2019). EGF-GH Axis in rat steatotic and non-steatotic liver transplantation from brain-dead donors. Transplantation 103 (7), 1349–1359. 10.1097/TP.0000000000002636 31241554

[B2] Atzori M. G. Ceci C. Ruffini F. Scimeca M. Cicconi R. Mattei M. (2022). The anti-vascular endothelial growth factor receptor 1 (VEGFR-1) D16F7 monoclonal antibody inhibits melanoma adhesion to soluble VEGFR-1 and tissue invasion in response to placenta growth factor. Cancers 14 (22), 5578. 10.3390/cancers14225578 36428669 PMC9688925

[B3] Avalos-de León C. G. Jiménez-Castro M. B. Cornide-Petronio M. E. Gulfo J. Rotondo F. Gracia-Sancho J. (2019). The effect of fibroblast growth factor 15 signaling in non-steatotic and steatotic liver transplantation from cardiocirculatory death. Cells 8 (12), 1640. 10.3390/cells8121640 31847428 PMC6952771

[B4] Barr T. Helms C. Grant K. Messaoudi I. (2016). Opposing effects of alcohol on the immune system. Prog. Neuro-Psychopharmacology Biol. Psychiatry 65, 242–251. 10.1016/j.pnpbp.2015.09.001 PMC4911891 26375241

[B5] Behrends M. Martinez-Palli G. Niemann C. U. Cohen S. Ramachandran R. Hirose R. (2010). Acute hyperglycemia worsens hepatic ischemia/reperfusion injury in rats. J. Gastrointest. Surg. 14, 528–535. 10.1007/s11605-009-1112-3 19997981 PMC2820661

[B6] Bobic S. Hox V. Callebaut I. Vinckier S. Jonckx B. Stassen J. M. (2014). Vascular endothelial growth factor receptor 1 expression in nasal polyp tissue. Allergy Eur. J. Allergy Clin. Immunol. 69 (2), 237–245. 10.1111/all.12277 24127643

[B7] Boccanegra B. Cappellari O. Mantuano P. Trisciuzzi D. Mele A. Tulimiero L. (2023). Growth hormone secretagogues modulate inflammation and fibrosis in mdx mouse model of duchenne muscular dystrophy. Front. Immunol. 14 (April), 1119888–1119916. 10.3389/fimmu.2023.1119888 37122711 PMC10130389

[B8] Boucher J. M. Clark R. P. Chong D. C. Citrin K. M. Wylie L. A. Bautch V. L. (2017). Dynamic alterations in decoy VEGF receptor-1 stability regulate angiogenesis. Nat. Commun. 8, 15699–15715. 10.1038/ncomms15699 28589930 PMC5467243

[B9] Bujaldon E. Cornide-Petronio M. E. Gulfo J. Rotondo F. Ávalos de León C. Negrete-Sánchez E. (2019). Relevance of VEGFA in rat livers subjected to partial hepatectomy under ischemia-reperfusion. J. Mol. Med. 97 (9), 1299–1314. 10.1007/s00109-019-01811-y 31254006 PMC6713699

[B10] Caicedo D. Díaz O. Devesa P. Devesa J. (2018). Growth hormone (GH) and cardiovascular system. Int. J. Mol. Sci. 19 (1), 290–340. 10.3390/ijms19010290 29346331 PMC5796235

[B11] Carnevale M. E. Lausada N. Juan de Paz L. Stringa P. Machuca M. Rumbo M. (2019). The novel N,N-Bis-2-Hydroxyethyl-2-Aminoethanesulfonic acid–gluconate–polyethylene glycol–hypothermic machine perfusion solution improves static cold storage and reduces ischemia/reperfusion injury in rat liver transplant. Liver Transplant. 25 (9), 1375–1386. 10.1002/lt.25573 31121085

[B12] Carrasco-Chaumel E. Roselló-Catafau J. Bartrons R. Franco-Gou R. Xaus C. Casillas A. (2005). Adenosine monophosphate-activated protein kinase and nitric oxide in rat steatotic liver transplantation. J. Hepatology 43 (6), 997–1006. 10.1016/j.jhep.2005.05.021 16085333

[B13] Casillas-Ramírez A. Micó-Carnero M. Sánchez-González A. Maroto-Serrat C. Trostchansky A. Peralta C. (2023). NO–IL-6/10–IL-1β Axis: a new pathway in steatotic and non-steatotic liver grafts from brain-dead donor rats. Front. Immunol. 14 (August), 1–27. 10.3389/fimmu.2023.1178909 PMC10427871 37593740

[B14] Ceccanti M. Mancinelli R. Tirassa P. Laviola G. Rossi S. Romeo M. (2012). Early exposure to ethanol or red wine and long-lasting effects in aged mice. A study on nerve growth factor, brain-derived neurotrophic factor, hepatocyte growth factor, and vascular endothelial growth factor. Neurobiol. Aging 33 (2), 359–367. 10.1016/j.neurobiolaging.2010.03.005 20382450

[B15] Costa R. Rodrigues I. Guardão L. Rocha-Rodrigues S. Silva C. Magalhães J. (2017). Xanthohumol and 8-prenylnaringenin ameliorate diabetic-related metabolic dysfunctions in mice. J. Nutr. Biochem. 45, 39–47. 10.1016/j.jnutbio.2017.03.006 28431322

[B16] Ding Bi S. Nolan D. J. Butler J. M. James D. Babazadeh A. O. Rosenwaks Z. (2010). Inductive angiocrine signals from sinusoidal endothelium are required for liver regeneration. Nature 468 (7321), 310–315. 10.1038/nature09493 21068842 PMC3058628

[B17] Falkevall A. Mehlem A. Folestad E. Ning F. C. Osorio-Conles Ó. Radmann R. (2023). Inhibition of VEGF-B signaling prevents non-alcoholic fatty liver disease development by targeting lipolysis in the white adipose tissue. J. Hepatology 78 (5), 901–913. 10.1016/j.jhep.2023.01.014 36717026

[B18] Fang F. Shi X. Brown M. S. Goldstein J. L. Liang G. (2019). Growth hormone acts on liver to stimulate autophagy, support glucose production, and preserve blood glucose in chronically starved mice. Proc. Natl. Acad. Sci. U. S. A. 116 (15), 7449–7454. 10.1073/pnas.1901867116 30910968 PMC6462072

[B19] Gulfo J. Rotondo F. Ávalos de León C. G. Cornide-Petronio M. E. Fuster C. Gracia-Sancho J. (2020). FGF15 improves outcomes after brain dead donor liver transplantation with steatotic and non-steatotic grafts in rats. J. Hepatology 73 (5), 1131–1143. 10.1016/j.jhep.2020.05.007 32422221

[B20] Kamada N. Calne R. Y. (1979). Orthotopic liver transplantation in the rat. Technique using cuff for portal vein anastomosis and biliary drainage. Transplantation 28 (1), 47–50. 10.1097/00007890-197907000-00011 377595

[B21] Kgosidialwa O. Hakami O. Zia-Ul-Hussnain H. M. Agha A. (2019). Growth hormone deficiency following traumatic brain injury. Int. J. Mol. Sci. 20 (13), 3323. 10.3390/ijms20133323 31284550 PMC6651180

[B22] Kivelä R. Bry M. Robciuc M. R. Räsänen M. Taavitsainen M. Silvola J. M. U. (2014). VEGF-B-Induced vascular growth leads to metabolic reprogramming and ischemia resistance in the heart. EMBO Mol. Med. 6 (3), 307–321. 10.1002/emmm.201303147 24448490 PMC3958306

[B23] Kreber L. A. Griesbach G. S. Ashley M. J. (2016). Detection of growth hormone deficiency in adults with chronic traumatic brain injury. J. Neurotrauma 33 (17), 1607–1613. 10.1089/neu.2015.4127 26414093 PMC5011623

[B24] Kwong A. J. Kim W. R. Lake J. R. Smith J. M. Schladt D. P. Skeans M. A. (2021). OPTN/SRTR 2019 annual data report: liver. Am. J. Transplant. 21 (S2), 208–315. 10.1111/ajt.16494 33595192

[B25] Lal N. Puri K. Rodrigues B. (2018). Vascular endothelial growth factor B and its signaling. Front. Cardiovasc. Med. 5 (April), 39–9. 10.3389/fcvm.2018.00039 29732375 PMC5920039

[B26] Lewis S. A. Cinco I. R. Doratt B. M. Blanton M. B. Hoagland C. Newman N. (2023). Chronic alcohol consumption dysregulates innate immune response to SARS-CoV-2 in the lung. eBioMedicine 97, 104812. 10.1016/j.ebiom.2023.104812 37793211 PMC10562860

[B27] Li R. Li Y. Yang X. Hu Y. Yu H. Li Y. (2022). Reducing VEGFB accelerates NAFLD and insulin resistance in mice via inhibiting AMPK signaling pathway. J. Transl. Med. 20 (1), 341–418. 10.1186/s12967-022-03540-2 35907871 PMC9338666

[B28] Li S. Hou G. Wang Y. Su X. Xue L. (2010). Influence of recombinant human growth hormone (RhGH) on proliferation of hepatocellular carcinoma cells with positive and negative growth hormone receptors *in vitro* . Tumori 96 (2), 282–288. 10.1177/030089161009600216 20572587

[B29] Li X. Kumar A. Zhang F. Lee C. Tang Z. (2012). Complicated Life, complicated VEGF-B. Trends Mol. Med. 18 (2), 119–127. 10.1016/j.molmed.2011.11.006 22178229

[B30] Mackowiak B. Fu Y. Maccioni L. Gao B. (2024). Alcohol-associated liver disease. J. Clin. Investigation 134 (3), e176345. 10.1172/JCI176345 PMC10836812 38299591

[B31] Melincovici C. S. Boşca A. B. Şuşman S. Mărginean M. Mihu C. Istrate M. (2018). Vascular endothelial growth factor (VEGF) – key factor in normal and pathological angiogenesis. Romanian J. Morphol. Embryology 59 (2), 455–467. 30173249

[B32] Micó-Carnero M. Casillas-Ramírez A. Sánchez-González A. Rojano-Alfonso C. Peralta C. (2022). The role of neuregulin-1 in steatotic and non-steatotic liver transplantation from brain-dead donors. Biomedicines 10 (5), 978. 10.3390/biomedicines10050978 35625715 PMC9138382

[B33] Micó-Carnero M. Rojano-Alfonso C. Álvarez-Mercado A. I. Gracia-Sancho J. Casillas-Ramírez A. Peralta C. (2020). Effects of gut metabolites and microbiota in healthy and marginal livers submitted to surgery. Int. J. Mol. Sci. 22 (1), 44. 10.3390/ijms22010044 33375200 PMC7793124

[B34] Micó-Carnero M. Rojano-Alfonso C. Álvarez-Mercado A. I. Gracia-Sancho J. Casillas-Ramírez A. Peralta C. (2021). Effects of gut metabolites and microbiota in healthy and marginal livers submitted to surgery. Int. J. Mol. Sci. 22 (1), 44–28. 10.3390/ijms22010044 PMC7793124 33375200

[B35] Nevzorova Y. A. Boyer-Diaz Z. Cubero F. J. Gracia-Sancho J. (2020). Animal models for liver disease – a practical approach for translational research. J. Hepatology 73 (2), 423–440. 10.1016/j.jhep.2020.04.011 32330604

[B36] ONT. 2023. Memoria de Actividad de Donación y Trasplante Hepático - España 2022.

[B37] Pan E. T. Yoeli D. Galvan N. T. N. Kueht M. L. Cotton R. T. O'Mahony C. A. (2018). Cold ischemia time is an important risk factor for post–liver transplant prolonged length of stay. Liver Transplant. 24 (6), 762–768. 10.1002/lt.25040 29476693

[B38] Qin Y. Tian Y.p. (2010). Exploring the molecular mechanisms underlying the potentiation of exogenous growth hormone on alcohol-induced fatty liver diseases in mice. J. Transl. Med. 8, 120–215. 10.1186/1479-5876-8-120 21087523 PMC2994817

[B39] Radic I. Mijovic M. Tatalovic N. Mitic M. Lukic V. Joksimovic B. (2019). Protective effects of whey on rat liver damage induced by chronic alcohol intake. Hum. Exp. Toxicol. 38 (6), 632–645. 10.1177/0960327119829518 30784321

[B40] Raissadati A. Tuuminen R. Dashkevich A. Bry M. Kivelä R. Anisimov A. (2017). Vascular endothelial growth factor-B overexpressing hearts are not protected from transplant-associated ischemia-reperfusion injury. Exp. Clin. Transplant. 15 (2), 203–212. 10.6002/ect.2016.0181 27588416

[B41] Ran T. Liu Y. Tang S. He Z. Munteanu C. R. (2016). Gastrointestinal spatiotemporal MRNA expression of ghrelin vs growth hormone receptor and new growth yield machine learning model based on perturbation theory. Sci. Rep. 6 (April), 30174–30214. 10.1038/srep30174 27460882 PMC4962052

[B42] Rossato F. A. Su Yu Mackey A. Ng Y. S. E. (2020). Fibrotic changes and endothelial-to-mesenchymal transition promoted by Vegfr2 antagonism alter the therapeutic effects of vegfa pathway blockage in a mouse model of choroidal neovascularization. Cells 9 (9), 2057–2121. 10.3390/cells9092057 32917003 PMC7563259

[B43] Rufinatscha K. Ress C. Folie S. Haas S. Salzmann K. Moser P. (2018). Metabolic effects of reduced growth hormone action in fatty liver disease. Hepatol. Int. 12 (5), 474–481. 10.1007/s12072-018-9893-7 30206761 PMC6208861

[B44] Sarmento-Cabral A. Del Rio-Moreno M. Vazquez-Borrego M. C. Mahmood M. Gutierrez-Casado E. Pelke N. (2021). GH directly inhibits steatosis and liver injury in a sex-dependent and IGF1-independent manner. J. Endocrinol. 248 (1), 31–44. 10.1530/JOE-20-0326 33112796 PMC7785648

[B45] Shen Z. Zhang Z. Wang X. Yang K. (2018). VEGFB-VEGFR1 ameliorates ang II-induced cardiomyocyte hypertrophy through Ca2+-mediated PKG I pathway. J. Cell. Biochem. 119 (2), 1511–1520. 10.1002/jcb.26311 28771828

[B46] Tateno C. Kataoka M. Utoh R. Tachibana A. Itamoto T. Asahara T. (2011). Growth hormone-dependent pathogenesis of human hepatic steatosis in a novel mouse model bearing a human hepatocyte-repopulated liver. Endocrinology 152 (4), 1479–1491. 10.1210/en.2010-0953 21303949

[B47] Trifunović S. Manojlović-Stojanoski M. Ristić N. Jurijević B. Š. Balind S. R. Brajković G. (2016). Effects of prolonged alcohol exposure on somatotrophs and corticotrophs in adult rats: stereological and hormonal study. Acta Histochem. 118 (4), 353–360. 10.1016/j.acthis.2016.03.005 27017477

[B48] Van Der Hoeven J. A. B. Lindell S. van Schilfgaarde R. Molema G. Ter Horst G. J. Southard J. H. (2001). Donor brain death reduces survival after transplantation in rat livers preserved for 20 HR. Transplantation 72 (10), 1632–1636. 10.1097/00007890-200111270-00009 11726822

[B49] Vatsalya V. Issa J. E. Hommer D. W. Ramchandani V. A. (2012). Pharmacodynamic effects of intravenous alcohol on hepatic and gonadal hormones: influence of age and sex. Alcohol. Clin. Exp. Res. 36 (2), 207–213. 10.1111/j.1530-0277.2011.01600.x 21797891 PMC3258349

[B50] Weiss S. Kotsch K. Francuski M. Reutzel-Selke A. Mantouvalou L. Klemz R. (2007). Brain death activates donor organs and is associated with a worse I/R injury after liver transplantation. Am. J. Transplant. 7 (6), 1584–1593. 10.1111/j.1600-6143.2007.01799.x 17430397

[B51] Wejman J. Pyzlak M. Szukiewicz D. Jarosz D. Tarnowski W. Szewczyk G. (2013). Thrombospondin and VEGF-R: is there a correlation in inflammatory bowel disease? Mediat. Inflamm. 2013, 908259–908310. 10.1155/2013/908259 PMC3732645 23970816

[B52] Wu X. Fan X. Miyata T. Kim A. Cajigas-Du Ross C. K. Ray S. (2023). Recent advances in understanding of pathogenesis of alcohol-associated liver disease. Annu. Rev. Pathology Mech. Dis. 18, 411–438. 10.1146/annurev-pathmechdis-031521-030435 PMC10060166 36270295

[B53] Zhang F. Zarkada G. Han J. Li J. Dubrac A. Ola R. (2018). Lacteal junction zippering protects against diet-induced obesity. Science 361 (6402), 599–603. 10.1126/science.aap9331 30093598 PMC6317738

[B54] Zhang Qi Lu S. Li T. Yu L. Zhang Y. Zeng H. (2019). ACE2 inhibits breast cancer angiogenesis via suppressing the VEGFa/VEGFR2/ERK pathway. J. Exp. Clin. Cancer Res. 38 (1), 173–212. 10.1186/s13046-019-1156-5 31023337 PMC6482513

[B55] Zhou W. Liu K. Zeng L. He J. Gao X. Gu X. (2022). Targeting VEGF-A/VEGFR2 Y949 signaling-mediated vascular permeability alleviates hypoxic pulmonary hypertension. Circulation 146 (24), 1855–1881. 10.1161/CIRCULATIONAHA.122.061900 36384284

[B56] Zhou Y. Zhang T. Zhang Y. Y. Xu J. Zhang Q. (2023). Expression and distribution of erythropoietin, vascular endothelial growth factor (VEGF) and VEGF receptor 2 in small intestine of yaks at different ages. Folia Morphol. 82 (3), 683–695. 10.5603/FM.a2022.0058 35692112

