# Supplementary figures and images for: Relevance of the GH-VEGFB/VEGFA axis in liver grafts from brain-dead donors with alcohol-associated liver disease
Source: Front Cell Dev Biol. 2025 Jan 7;12:1455258. doi: 10.3389/fcell.2024.1455258 (PMC11747040; doi:10.3389/fcell.2024.1455258)

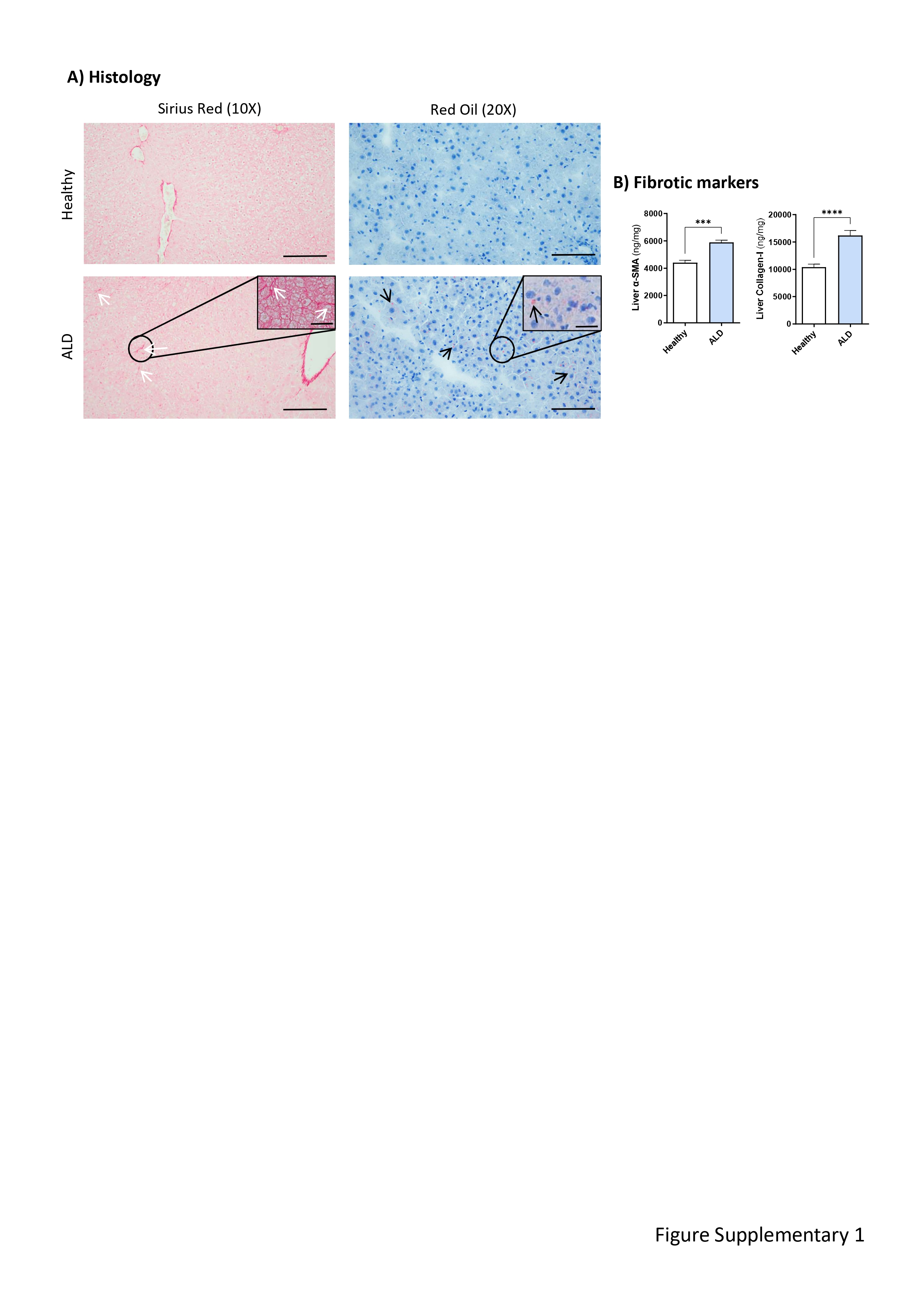

Supplement: Supplementary file 1 [file Image1.jpeg]

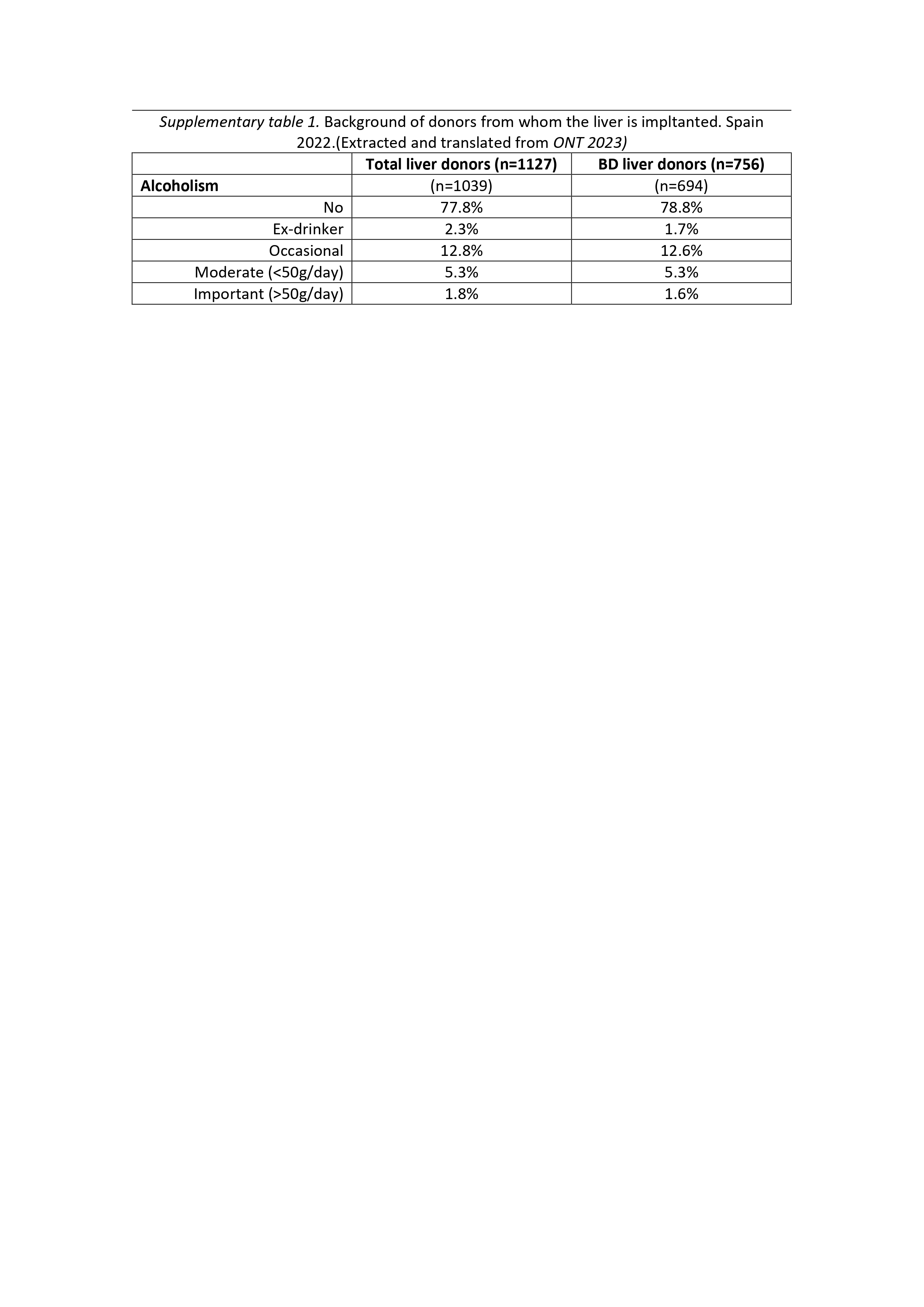

Supplement: Supplementary file 3 [file Supplementaryfile1.jpeg]
