# Supplementary material for: Relevance of the GH-VEGFB/VEGFA axis in liver grafts from brain-dead donors with alcohol-associated liver disease
Source: Front Cell Dev Biol. 2025 Jan 7;12:1455258. doi: 10.3389/fcell.2024.1455258 (PMC11747040; doi:10.3389/fcell.2024.1455258)
Supplement: Supplementary file 2 [file Supplementaryfile2.pdf]

Supplementary table 2. Transplant percentage of removed livers according to donor characteristics. Spain 2013-2022. (Extracted and translated from *ONT 2023)*

	**Total livers removed**	**% transplant**	**p***
**Alcoholism**			<0.001
No	10277	74.7%	
Ex-drinker	278	62.7%	
Moderate (<50g/day)	737	65%	
Important (>50g/day)	409	59.4%	
